# The association between the preoperative serum levels of lipocalin-2 and matrix metalloproteinase-9 (MMP-9) and prognosis of breast cancer

**DOI:** 10.1186/1471-2407-12-193

**Published:** 2012-05-28

**Authors:** Hyuna Sung, Ji-Yeob Choi, Sang-Ah Lee, Kyoung-Mu Lee, Sohee Han, Sujee Jeon, Minkyo Song, Yunhee Lee, Sue K Park, Keun-Young Yoo, Dong-Young Noh, Sei-Hyun Ahn, Daehee Kang

**Affiliations:** 1 Department of Biomedical Sciences, Seoul National University College of Medicine, Seoul, Korea; 2 Department of Preventive Medicine, Kangwon National University School of Medicine, Kangwon, Korea; 3 Department of Environmental Health, Korea National Open University, Seoul, Korea; 4 Department of Preventive Medicine, Seoul National University College of Medicine, Seoul, Korea; 5 Department of Molecular Medicine and Biopharmaceutical Sciences, Graduate School of Convergence Science and Technology and College of Medicine or College of Pharmacy, Seoul National University, Seoul, Korea; 6 Cancer Research Institute, Seoul National University College of Medicine, Seoul, Korea; 7 Department of Surgery, Seoul National University College of Medicine, Seoul, Korea; 8 Department of Surgery, University of Ulsan College of Medicine and Asan Medical Center, Seoul, Korea

## Abstract

**Background:**

Although a number of experimental studies have suggested the role of lipocalin-2 (LCN2) and matrix metalloproteinase-9 (MMP-9) in breast cancer progression, limited numbers of epidemiological studies have examined the relationship between the levels of lipocalin-2 and MMP-9 and breast cancer survival.

**Methods:**

Preoperative serum levels of lipocalin-2 and MMP-9 were measured in 303 breast cancer patients and 74 healthy controls recruited between 2004 and 2007. We examined the association between lipocalin-2 and MMP-9 levels and disease-free survival (DFS) using Cox proportional hazard regression model.

**Results:**

The serum levels of lipocalin-2 and MMP-9 were not significantly different between patients and controls (*P* > 0.05). Elevated lipocalin-2 and MMP-9 levels were associated with reduced DFS of breast cancer ( *P*_trend_ = 0.029 and *P*_trend_ = 0.063, respectively). When lipocalin-2 and MMP-9 levels were categorized based on the combined risk score, patients with higher levels of both lipocalin-2 and MMP-9 exhibited poor DFS compared to patients with lower levels (*P*_trend_ = 0.004). Furthermore, these effects were profound in patients with BMI less than 25 kg/m^2^ (adjusted hazard ratio (aHR), 3.17; 95% confidence intervals (CI), 1.66-6.06, *P*_trend_ < 0.001) or lymph-node negative breast cancer (aHR, 5.36; 95% CI, 2.18-13.2, *P*_trend_ < 0.001).

**Conclusions:**

Our study suggests that the elevated levels of lipocalin-2 and MMP-9 are associated with reduced breast cancer survival, particularly in patients with lower BMI and lymph-node negative breast cancers.

## Background

While breast cancer incidence has increased over the 30–40 years, mortality has remained stable or has even decreased in the last 10–15 years, probably contributed to earlier detection and improved treatment strategy as well [1,2]. Considering the substantial increase in breast cancer survivors, to identify prognostic factors associated with recurrence and survival is more important today ever than before after primary treatment.

Although commonly available prognostic factors include pathology criteria such as lymph-node status, tumor size, histologic grade and estrogen receptor (ER) status, these factors do not predict accurately exact clinical outcome probably due to heterogeneity of breast cancers. While numerous studies support the prognostic relevance of circulating tumor marker such as CA 15–3, CA 27, CA 29, CEA and tissue based multiparameter gene expression assays, it is not yet sufficient to support routine use in clinical practice [3].

Lipocalin-2, also known as neutrophil gelatinase-associated lipocalin (NGAL), is a 25 kDa secretary glycoprotein belonging to the lipocalin superfamily to act as transporter of small hydrophobic substances [4]. Initially known only as an antibacterial factor of innate immunity, lipocalin-2 has been suggested to participate in diverse biological processes such as, inflammation, acute organ damage, lipid metabolism, and cancer development. Particularly, lipocalin-2 has gained attention as a potential biomarker and a modulator of several types of human cancers, thus a potential therapeutic target as well [5]. Although the mechanisms are not clearly elucidated, several studies suggest possible mechanisms underlying the role of lipocalin-2 in mammary tumor initiation and progression [6].

Human lipocalin-2 was originally isolated in the complex with matrix metalloproteinase-9 (MMP-9), a zinc dependent proteolytic enzyme involved in the degradation of many different constituents of basement membranes and subsequent remodeling of extracellular matrix [7,8]. In preclinical model and in clinical samples, it was demonstrated that lipocalin-2 could protect the degradation of MMP-9 by forming the lipocalin-2/MMP-9 complex, which can enhance the enzymatic activity of MMP-9 and facilitate the tumor growth through promoting its invasion of adjacent tissues or metastasis to distal organs [9-11]. Not only the key roles of MMP-9 in tumorigenesis but also their characteristics of being secreted into the blood stream have inspired many researchers to evaluate the associations between circulating level of MMP-9 and clinicopathological characteristics of breast cancers. However, majority of these studies were merely designed to investigate the correlation between clinical characteristics or disease status and circulating levels of MMP-9.

Along with the previous report that the enzymatic activity of urinary MMP-9/lipocalin-2 complex is detected in the urine of breast cancer patients but not in healthy control [11], several studies have suggested that circulating MMP-9, lipocalin-2 and MMP-9/lipocalin-2 complex could serve as a diagnostic and/or a prognostic biomarker in a variety of diseases with different suggested mechanisms [12-15]. In breast cancer, only one study has reported the positive association between the serum level of lipocalin-2/MMP-9 complex and disease status [16]. However, no other prior studies have examined the relationship between the circulating level of lipocalin-2 and MMP-9 and their combined effects on the prognosis of breast cancer.

Given the findings that both lipocalin-2 and MMP-9 are mainly secreted into the normal breast ducts and by neutrophils that have infiltrated into the tumor sites [17], we hypothesized that the serum levels of lipocalin-2 and MMP-9 might represent the aggressiveness of breast cancer and might be an independent prognostic factor of breast cancer survival. In the present study, we examined the association between preoperative serum levels of lipocalin-2 and MMP-9 and disease-free survival (DFS) and determined the potential of both proteins as noninvasive biomarkers in predicting the recurrence of breast cancer.

## Methods

### Study population

A total of 1,899 histologically confirmed incident breast cancer cases were recruited in the Seoul National University Hospital and Asan Medical Center between 2004 and 2007 [18]. Healthy women were recruited from a general hospital to visit for a regular health check-up in the same catchment area for breast cancer cases. After excluding the patients with history of cancer, age frequency matched women (*N* = 74) were selected and included in the analysis. At the time of interview, before any adjuvant chemotherapy and/or surgery, peripheral blood were collected and processed for the DNA extraction and serum separation. For the 925 of the patients, peripheral blood were collected into 10-ml serum storage tubes and were centrifuged at 3000 g for 10 minutes at room temperature. Serum were stocked in 0.3-ml aliquots in cryovials and stored at −80°C until the time of measurement.

After excluding the subjects with inadequate follow-up information, about 40% (*N* = 370) of subjects were selected. A retrospective chart review was used to collect clinical information and pathologic features including cancer stage based on 6th AJCC classification, tumor size, lymph-node invasion, distant organ metastasis, histologic grade, nuclear grade, estrogen receptor (ER) and progesterone receptor (PR) status, nuclear grade, histological grade, surgical treatment and medical adjuvant therapy (adjuvant chemotherapy, radiation therapy, and hormone receptor therapy). Those with prehistory of cancer, multi-cancer at diagnosis, distant organ metastasis at diagnosis, and *in situ* breast cancer were additionally excluded. Thus, a total of 303 invasive ductal carcinoma cancer patients diagnosed with stage I-IIIC who underwent curative resection were included for final analysis.

Informed consents were received from every patient when the questionnaire was administered. The study design was approved by the Committee on Human Research of Seoul National University Hospital (IRB No. H-0503-144-004).

### Serum lipocalin-2 and MMP-9 assays

The serum levels of proteins were determined using the Quantikine® Human Lipocalin-2/NGAL Immunoassay kit (R&D Systems, Minneapolis, MN, USA) and Human MMP-9 ELISA Kit (Bender MedSystem, Vienna, Austria) according to the manufacturer’s instruction. Reproducibility within the assay was evaluated with an intra-assay coefficient of variation of 3.7% for lipocalin-2 and 7.3% for MMP-9, respectively. The specificity was confirmed by testing its cross-reactivity with human recombinant COX-2, lipocalin-1, MMP-9 and mouse recombinant lipocain-2. No significant cross-reactivity or interference was observed other than recombinant human MMP-9/NGAL complex shows approximately 0.3% cross reactivity in this assay. The minimum detectable dose (MDD) was defined as the analyte concentration resulting in an absorbance significantly higher than that of the dilution medium (mean plus 2 standard deviations (SD)). The mean MDD of lipocalin-2 and MMP-9 was 0.012 ng/ml and 0.05 ng/ml, respectively (mean of independent assays).

### Statistical analyses

The serum levels of lipocalin-2 and MMP-9 between cases and controls were compared by non-parametric Wilcoxon signed rank test and Student’s t-test. The distributions of lipocalin-2 and MMP-9 according to demographic factors and clinicopathological variables were compared by using Mantel-Haenszel chi-square test for categorical variables and Pearson correlation coefficients test for continuous variables. Since the levels of lipocalin-2 and MMP-9 were not normally distributed (the Kolmogorov–Smirnov test, *P* < 0.10), log square transformed values were used for correlation tests and survival analyses.

DFS was defined as the time from date of surgery to the date of the first locoregional recurrence, first distant metastasis, 2^nd^ primary cancer or death from any cause. Patients known to be alive with no evidence of disease were censored at the last follow-up date.

Cox’s proportional hazard regression models were used to estimate hazard ratios (HR) and 95% confidence intervals (CI). The proportional hazard assumption of the Cox model was examined by graphic evaluation of Schoenfeld residual plot. The multivariate model included TNM stage (I, IIA-IIB, and IIIA-IIIC), ER status, and radiation therapy (yes and no). We included BMI (< 25 kg/m2 and ≥ 25 kg/m^2^) as well due to the significant association between lipocalin-2 and BMI groups. Other covariates considered but not included the final model were age (≤ 39, 40–49, and ≥ 50), menopausal status, lymph-node status, tumor size, histologic grade (I-II and III), nuclear grade (I-II and III), PR status, adjuvant chemotherapy and hormone receptor therapy. Because these variables did not alter HRs significantly after adjusting for other covariates (statistical significance was set at *P* < 0.05) and the full model adjusted for all covariates made no substantial difference to the results compared to the reduced model. We conducted analysis with lipocalin-2 and MMP-9 as continuous variables and as categorical variables in tertiles based on the distributions of cases.

To further assess the combined effects of both markers, we generated an equation, combined risk score = exp [(0.054 x log square transformed values of lipocalin-2) + (0.024 x log square transformed values of MMP-9)], based on the linear regression model using the coefficients of each marker determined by the multivariate model including each marker (continuous) and adjustment variables. The range of combined score was 1.092 to 14.276. The subjects were categorized into tertiles based on the distribution of the combined score: low (1.092-3.463), medium (3.464-4.997) and high (4.998-14.276) score groups. Wald *P* values for trend were computed by treating the tertiles or combined score groups as ordinal variable.

Additionally, stratified analyses were performed according to age group, menopausal status, BMI group, stage, tumor size, lymph-node status and ER/PR status. We tested for heterogeneity between strata by including product term of marker and stratification variable of interest in regression model.

To evaluate the predictive accuracy of serum biomarkers, receiver operating characteristic (ROC) curve and the area under the curve (AUC) were analyzed. In addition, to assess the internal validity of our model, we used k-fold cross-validation method using the entire dataset as both for development and validation of the model [19].

All statistical procedures were conducted using SAS version 9.2 (SAS Institute, Cary, NC) and STATA version 11.2 (Statacorp, College Station, TX). All *P* values reported were two-sided.

## Results

**Figure 1:**
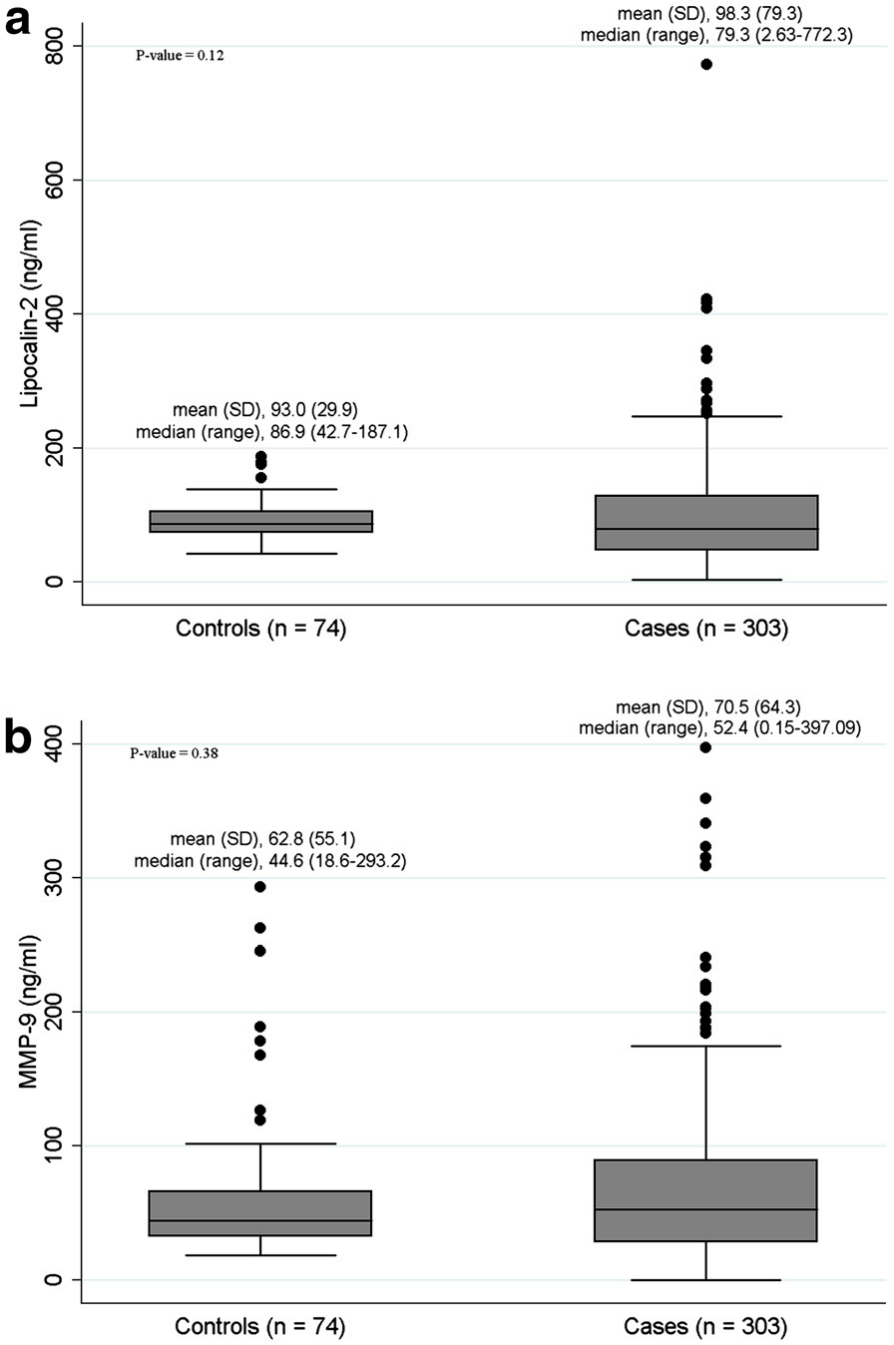
**Serum levels of (a) lipocalin-2 and (b) MMP-9 depicted as box-plots.** The levels of both markers were compared between breast cancer cases ( *N* = 303) and age and sex matched healthy controls ( *N* = 74). Lines inside boxes represent the median value and each box represents the interquartile range. Lines extend to minimum and maximum values excluding extreme values (circles). *P*-value was calculated from non-parametric Wilcoxon signed rank test.

The baseline characteristics of 303 breast cancer cases and 74 age and sex matched controls were described in Additional file 1: Table S 1. The distributions of age and BMI were not different between cases and controls. Figure 1 shows the distribution of lipocalin-2 and MMP-9 in cases and controls. The levels of both markers were not significantly different between groups although the means of lipocalin-2 and MMP-9 of cases were slightly higher than those of controls (98.8 ± 81.7 *vs*. 93.0 ± 29.9 ng/ml for lipocalin-2 and 70.5 ± 64.3 *vs.* 62.8 ± 55.1 ng/ml, respectively).

After a median follow-up of 4.2 years (range, 0.2-5.3 years) there were 87 DFS events including 10 deaths from any cause among all 303 patients. Table 1 summarized univariate and multivariate–adjusted HRs for DFS by patients' characteristics. Most patients were premenopausal (63.3%) and had BMI less than 25 kg/m^2^ (75.6%). In multivariate analysis, TNM stage, ER status and radiation therapy were remained as independent and significant prognostic factors for DFS (*P* < 0.05).

**Table 1:** Hazard ratios for disease-free survival of breast cancer

	**All patients (*N* = 303)**		**Event (*N* = 87)**		**Crude HR(95% CI)**	***P***	**Adjusted HR**^**a**^**(95% CI)**	***P***
	***N***	**%**	***N***	**%**				
F/U duration (median), days	67-1930 (1540)		67-1930 (1161)					
Age, mean (SD)	46.6 (10.7)		46.5 (11.4)		0.99 (0.79-1.23)	0.897	1.00 (0.98-1.02)	0.911
< 39	81	26.7	25	28.7	1.00		1.00	
40-49	108	35.6	29	33.3	0.85 (0.50-1.46)	0.557	1.21 (0.68-2.16)	0.525
50 ≤	114	37.7	33	37.9	0.83 (0.49-1.41)	0.496	0.97 (0.54-1.76)	0.926
Menopausal status^b^								
Premenopausal	191	63.3	54	62.1	1.00		1.00	
Postmenopausal	111	36.8	33	37.9	1.02 (0.66-1.58)	0.915	1.01 (0.62-1.64)	0.982
BMI, mean (SD)	23.3 (3.17)		23.5 (3.39)					
< 25 kg/m^2^	229	75.6	62	71.3	1.00		1.00	
≥ 25 kg/m^2^	74	24.4	25	28.7	1.11 (0.70-1.77)	0.665	1.10 (0.66-1.81)	0.723
TNM stage								
I	115	38	18	20.7	1.00		1.00	
IIA -IIB	123	40.5	36	41.4	1.89 (1.06-3.37)	0.031	1.46 (0.78-2.73)	0.242
IIIA -IIIC	65	21.5	33	37.9	3.97 (2.20-7.13)	<.0001	4.41 (2.27-8.57)	<.0001
Tumor size								
< 2 cm	159	52.5	26	29.9	1.00		1.00	
≥ 2 cm	144	47.5	61	70.1	2.78 (1.74-4.44)	<.0001	2.42 (1.46-4.02) ^c^	<.0001
Lymph node status								
Negative	170	56.1	40	46	1.00		1.00	
Positive	133	43.9	47	54	1.54 (1.27-1.86)	<.0001	1.47 (0.93-2.31) ^d^	0.092
Histologic grade^b^								
I-II	160	55.4	32	39	1.00		1.00	
III	129	44.6	50	61	2.11 (1.35-3.30)	0.001	1.36 (0.81-2.28)	0.248
Nuclear grade^b^								
I-II	145	54.6	33	39.8	1.00		1.00	
III	133	45.4	50	60.2	1.96 (1.26-3.06)	0.003	1.16 (0.70-1.95)	0.564
ER status								
Positive	173	57.1	36	41.4	1.00		1.00	
Negative	130	42.9	51	58.6	2.40 (1.56-3.70)	<.0001	2.34 (1.46-3.75)	<.0001
PR status								
Positive	159	52.5	33	37.9	1.00		1.00	
Negative	144	47.5	54	62.1	2.19 (1.41-3,.39)	0.001	1.45 (0.79-2.65)	0.235
Adjuvant chemotherapy^b^								
Yes	216	72.7	74	87.1	1.00		1.00	
No	81	27.3	11	12.9	0.34 (0.17-0.65)	0.001	0.61 (0.26-1.41)	0.249
Radiation therapy^b^								
Yes	184	61.7	49	57.7	1.00		1.00	
No	114	38.3	36	42.4	1.26 (0.81-1.94)	0.310	2.08 (1.24-3.49)	0.006
Hormone receptor therapy^b^								
Yes	198	66.2	41	47.7	1.00		1.00	
No	101	33.8	45	52.3	2.65 (1.73-4.06)	<.0001	2.03 (0.90-4.60)	0.089

^a^ Adjusted for BMI (< 25 and ≥25 kg/m^2^), TNM stage (I, II, and III), ER status (positive and negative) and radiation therapy (yes and no).

^b^ Due to missing information, total frequency is less than 303.

^c^ Adjusted for BMI (< 25 and ≥25 kg/m^2^), lymph node status (yes and no), ER status (positive and negative) and radiation therapy (yes and no).

^d^ Adjusted for BMI (< 25 and ≥25 kg/m^2^), tumor size (< 2 and ≥2 cm^2^), ER status (positive and negative) and radiation therapy (yes and no).

HR, hazard ratio; CI, confidence interval; BMI, body mass index; ER, estrogen receptor; PR, progesterone receptor.

There was significant correlation between serum levels of lipocalin-2 and MMP-9 (r_pearson_^2^ = 0.267; *P* < 0.001). Table 2 shows the distributions of patients’ characteristics according to tertiles of lipocalin-2 and MMP-9. Women with higher lipocalin-2 levels were more likely to be heavier compared with women with low lipocalin-2 levels (*P*_trend_ = 0.054). No other significant association was found between lipocalin-2 and MMP-9 and clinicopathological characteristics.

**Table 2:** The distributions of demographic and clinicophathological characteristics according to tertile of lipocalin-2 and MMP-9 in all patients

	**Lipocalin-2 (ng/ml)**				**MMP-9 (ng/ml)**			
	**t1**	**t2**	**t3**					***P***_**trend**_^**a**^	**t1**	**t2**	**t3**	***P***_**trend**_^**a**^
	**(2.63-59.03)**	**(59.03-109.65)**	**(109.65-772.34)**		**(0.15-34.42)**	**(34.42-80.36)**	**(80.36-397.09)**					
Age, mean (SD)	46.5 (11.6)	47.3 (10.6)	45.9 (9.9)	0.67^b^	48.2 (10.5)	44.4 (9.8)	47.1 (11.5)	0.58^b^
Premenopausal status, %	65.0	60.4	64.4	0.93	53.0	72.6	64.0	0.11
BMI < 25 kg/m^2^, %	82.0	74.5	70.3	0.05	77.0	78.4	71.3	0.35
TNM stage, %								
I	32.0	40.2	41.6	0.59	35.0	46.1	32.7	0.72
IIA –IIB	48.0	39.2	34.7		39.0	35.3	47.5	
IIIA –IIIC	20.0	20.6	23.8		26.0	18.6	19.8	
Tumor size < 2cm, %	46.0	53.9	57.4	0.11	47.0	62.8	47.5	0.95
Lymph-node negative, %	57.0	54.9	56.4	0.94	55.0	60.8	52.5	0.72
Histologic grade I-II, %	48.9	61.2	55.7	0.36	48.4	62.2	55.2	0.35
Nuclear grade I-II, %	51.6	58.0	54.1	0.73	52.1	57.0	54.6	0.72
ER positive, %	56.0	59.8	55.5	0.94	53.0	61.8	56.4	0.63
PR positive, %	51.0	54.9	51.5	0.95	48.0	58.8	50.5	0.73
Adjuvant chemotherapy Yes, %	75.8	66.7	75.8	0.99	74.2	70.0	74.0	0.98
Radiation therapy Yes, %	62.2	58.0	65.0	0.69	67.7	60.6	57.0	0.12
Hormone receptor therapy Yes, %	63.6	69.0	66.0	0.73	62.6	70.0	66.0	0.61

^a^ Mantel-Haenszel chi-square test.

^b^ Pearson correlation coefficients test.

Table 3 shows the stage-, ER status-, BMI, and radiation therapy-adjusted HRs for DFS by lipocalin-2 and MMP-9 levels in overall patients. The elevated serum levels of lipocalin-2 and MMP-9 were significantly associated with poor DFS, respectively and in combination. The adjusted HR for the 3 categories of lipocalin-2 were 1.96 (95% CI, 1.12-3.43) for the second tertile and 1.93 (95% CI, 1.07-3.47) for the third tertile compared with first tertile as reference group (*P*_trend_ = 0.029). The similar but marginally significant relationship was observed for the association between MMP-9 and DFS (adjusted HR for third *vs.* first tertile 1.70; 95% CI 0.97-2.99; *P*_trend_ = 0.063).

**Table 3:** Multivariate–adjusted hazard ratios for disease-free survival of lipocalin-2 and MMP-9

		**No. of total patients (*N*=303)**	**No. of events (*N*=87)**	**Adjusted HR**^**a**^** (95% CI)**	***P***
Lipocalin-2	continuous			1.06 (1.02-1.09)	0.001
(ng/ml)	t1	100	22	1.00 (reference)	
	t2	102	35	1.96 (1.12-3.43)	0.018
	t3	101	30	1.93 (1.07-3.47)	0.028
	*P*_trend_			1.36 (1.03-1.79)	0.029
MMP-9	continuous			1.02 (0.99-1.06)	0.136
(ng/ml)	t1	100	22	1.00 (reference)	
	t2	102	34	1.95 (1.12-3.39)	0.018
	t3	101	31	1.70 (0.97-2.99)	0.065
	*P*_trend_			1.28 (0.99-1.67)	0.063
Combined score^b^	low	101	24	1.00 (reference)	
	medium	100	28	1.55 (0.88-2.74)	0.130
	high	102	35	2.22 (1.29-3.84)	0.004
	*P*_trend_			1.49 (1.14-1.95)	0.004

^a^ Adjusted for BMI (< 25 and ≥25 kg/m^2^), TNM stage (I, II, and III), ER status (positive and negative) and radiation therapy (yes and no).

^b^ The subjects were categorized into three groups based on the tertiles of combined score: low (1.092-3.463), medium (3.464-4.997) and high (4.998-14.276) score groups.

Furthermore, we evaluated the combined effect of lipocalin-2 and MMP-9 on DFS. When the patients were classified into three groups based on the combined scores, patients in the high score group had exhibited significantly worse DFS compared to those belonging to low score group (adjusted HR 2.22; 95% CI, 1.29-3.84; *P*_trend_ = 0.004).

**Figure 2:**
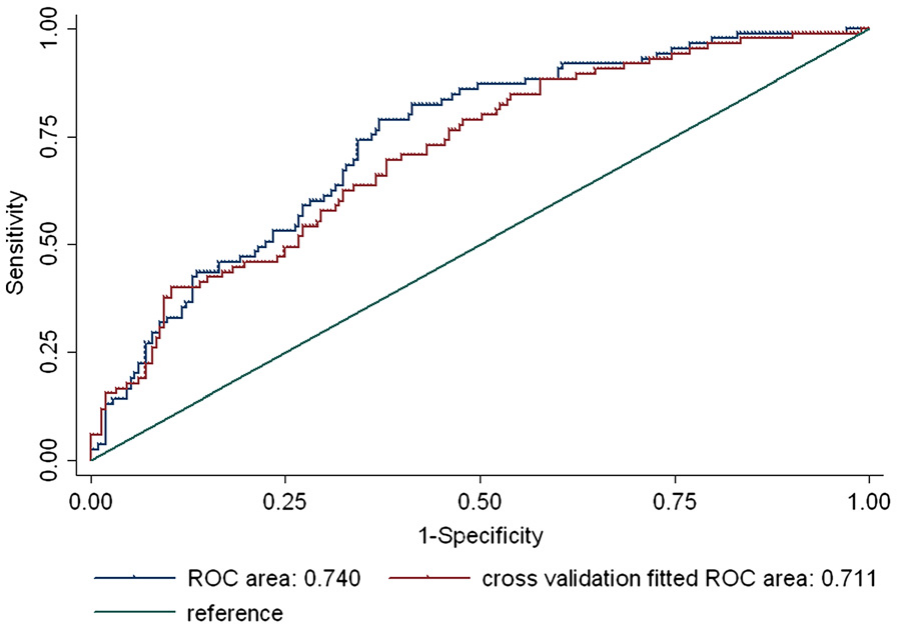
**The ROC curve of the final model.** The naïve model was fitted with Cox proportional hazard regression model using combined score (continuous) adjusted for BMI (< 25 and ≥25 kg/m^2^), TNM stage (I, II, and III), ER status (positive and negative) and radiation therapy (yes and no). The prediction error fitted ROC was estimated using 10-fold cross validation. Among the 303 patients, 298 subjects were used in ROC analysis since the subjects with missing values of any covariates were excluded.

The ability of combined score to predict breast cancer survival was verified using an analysis of ROC curve (Additional file 1: Table S 2). The AUC of combined score of both markers was 0.568 (95% CI, 0.50-0.64). In addition, the AUC fitted with the model including combined score and other covariates was 0.740 (95% CI, 0.68-0.80). Furthermore, the internal validity was evaluated using 10-fold cross validation. The AUC of prediction error (PE) fitted model was 0.711 (95%, 0.65-0.78) which was slightly less than that of naïve model Figure 2.

The adverse association between combined scores and DFS seemed to be even stronger in certain subgroups including women with a BMI less than 25 km/m^2^ and women whose tumors were lymph-node negative (Table 4). The adjusted HR of high score group was 3.17 compared with low score group (95% CI, 1.66-6.06; *P* < 0.001; *P*_trend_ < 0.001), but no significant association was found in patients with a BMI more than 25 km/m^2^ (*P*_interaction_ = 0.031). A more significant and profound association was found in lymph-node negative patients with an adjusted HR of 5.36 (95% CI, 2.18-13.2; *P* < 0.001; *P*_trend_ < 0.001) than in lymph-node positive patients (*P*_interaction_ = 0.094). However, the associations between the score group and DFS were not different by age group, tumor size, histologic grade, nuclear grade, and hormone receptor status (*P*_interaction_ > 0.10) (data not shown).

**Table 4:** Hazard ratios for disease-free survival and the combined risk scores of serum lipocalin-2 and MMP-9 levels according to BMI and lymph node status

	**Combined score **^**a**^	**No. of total patients (*N*=303)**	**No. of events (*N*=87)**	**Adjusted HR**^**b**^**(95% CI)**	***P***	***P***_**interaction**_
BMI < 25	t1	81	17	1.00 (reference)		0.031
*N*=229	t2	77	20	1.74 (0.88-3.42)	0.110	
	t3	71	25	3.17 (1.66-6.06)	<.0001	
	*P*_trend_			1.78 (1.29-2.46)	<.0001	
BMI ≥ 25	t1	20	13	1.00 (reference)		
*N* =74	t2	23	15	0.92 (0.27-3.08)	0.886	
	t3	31	21	0.92 (0.32-2.63)	0.881	
	*P*_trend_			0.96 (0.57-1.62)	0.891	
LN –	t1	57	9	1.00 (reference)		0.094
*N* =170	t2	55	12	3.28 (1.19-9.07)	0.022	
	t3	58	19	5.36 (2.18-13.2)	<.0001	
	*P*_trend_			2.21 (1.45-3.35)	<.0001	
LN +	low	44	29	1.00 (reference)		
*N* =133	medium	45	29	1.31 (0.64-2.68)	0.462	
	high	44	28	1.43 (0.69-2.95)	0.335	
	*P*_trend_			1.20 (0.84-1.71)	0.328	

^a^ The subjects were categorized into three groups based on the tertiles of combined score: low (1.092-3.463), medium (3.464-4.997) and high (4.998-14.276) score groups.

^b^ Adjusted for BMI (< 25 and ≥ 25 kg/m^2^), TNM stage (I, II, and III), ER status (positive and negative) and radiation therapy (Yes and No). For stratified analysis by BMI group, the stratification variable of interest (BMI) was not included as covariate in the model.

LN –, lymph-node negative; LN +, lymph-node positive.

## Discussion

In the present study, we found that patients with elevated lipocalin-2 or MMP-9 levels at diagnosis had poorer DFS than patients with low lipocalin-2 or MMP-9 levels. Women with a high level of lipocalin-2 (> 59.03 ng/ml) and MMP-9 (> 34.42 ng/ml) had significantly increased risk for recurrence or death than women with a low level of each protein. In addition, patients with both markers in high levels showed significantly poor DFS, especially in the women with low BMI or lymph-node negative breast cancer.

Although Noh *et al.* suggested the lymphovascular invasion, poorer histologic grade and higher nuclear grade are associated with poor DFS in Korean breast cancer cases in recently published paper [20], histologic grade and nuclear grade were not independently associated with DFS in our subjects. There could be several reasons for this varied survival profile such as relatively shorter follow-up period of our study, different baseline characteristics of study population and definition of outcome of both studies, and uncontrolled bias from method of tumor assessment.

Several studies investigated the serum level of lipocalin-2 and MMP-9 not only in patients with diseases but also in healthy controls. The previous observational studies have shown that the mean or median baseline value of lipoclalin-2 is highly contradictory dependent on the study design and assay method [21,22]. The normal range of lipocalin-2 measured through our study (42.7-187.1 ng/ml) was comparable to the range suggested by Porta *et al.* (47–177 ng/ml ) [23,24]. However, Choi *et al.* suggested the normal range as 43.8 ± 27.8 ng/ml which is much lower than our data (93.0 ± 29.9 ng/ml) [25].

Similarly the previous reports on the serum level of MMP-9 in healthy controls are highly contradictory [16,26]. Although with a small sample size, Provatopoulou *et al.* demonstrated a marked increase in serum concentration of lipocalin-2 and MMP-9 for women with invasive ductal carcinoma compared with the healthy controls. This inconsistency across the studies might be caused mainly due to the method of sample collection (timing, storage, and preparation etc.), detection method and uncontrolled potential confounding factors related to patients’ characteristics. Furthermore, several factors related to the biochemical characteristics of each marker could have disturbed the concentrations of serum proteins such as the coagulation and fibrinolysis factor and leukocyte degraulation [27]. In addition, given the sample size of our study, the standard deviation is too large, especially for both markers measured in cases, to obtain enough power to compare the concentrations between cases and controls. Thus our results should be interpreted with cautions and need to be confirmed in larger sample size. For this reason, we evaluated the variables based on the distribution of cases samples rather than to define the normal range of lipocalin-2.

In a previous study using tissue microarray containing samples from 207 breast cancer patients, elevated expression of lipocalin-2 correlates with some indicators of a phenotype severity including a low ER/PR expression, a low grade of differentiation, the presence of lymph-node metastases and a high Ki-67 proliferation index, and overexpressed lipocalin-2 is associated with poor DFS consistent with our results using serum [17,28,29].

The relationship between high levels of lipocalin-2 being detected in clinical samples of different tumor types, especially in epithelial origin tumors, such as esophageal [30], gastric [31], ovarian [32], pancreatic cancers [33] with clinical outcomes are being published. However, the precise source of serum lipocalin-2 has remained controversial. It is plausible that the overexpressed lipocalin-2 in tumor tissue released into the circulation may contribute to the elevated systematic level of lipocalin-2 supported by the direct correlation between elevated serum levels of lipocalin-2 and strong immunostaining grade in gastric tumor cells [34]. In addition, as an acute-phase protein, lipocalin-2 may be released into the plasma from activated neutrophils, macrophages, and other immune cells in various inflammatory conditions [35,36].

Recent findings suggest possible mechanisms underlying lipocalin-2 function in tumorigenesis, such as promoting the epithelial-mesenchymal transition (EMT) [29], regulating the iron homeostasis in cancer cells, affecting on steroid-dependent tumor growth and modulating MMP-9 activity [6]. When lipocalin-2 is complexed with MMP-9, there is enhancement of the active MMP-9 pool [9]. The cooperative role between lipocalin-2 and MMP-9 in breast cancer progression prompted us to investigate the concomitant presence of MMP-9 and we found the positive correlation between lipocalin-2 and MMP-9 in serum from breast cancer patients. Although several reports suggest that elevated serum levels of MMP-9 occur in serum and plasma of breast cancer patients [37-42], correlation with current clinical parameters and association with clinical outcome are still controversial mainly due to different types of samples being assayed and sampling procedures [27].

Furthermore, we observed the prognostic value is further intensified in certain subgroups. We found stronger and significant association in patients with a BMI less than 25 kg/m^2^. When we examined this association with a median value of BMI, the similar pattern was also observed although *P* for interaction was marginally significant. This finding may be partly explained by the fact that, among patients with a BMI less than 25 kg/m^2^, there was significant difference in lipocalin-2 levels between patients alive without recurrence and patients who had recurrence (*P* < 0.05). Given that obesity at diagnosis of breast cancer has been associated with an increased risk of recurrence and death [43,44], it is possible that the impact of lipocalin-2 level and/or combined score on DFS could only be demonstrated in the lower BMI group, with these markers being independent prognostic factors of breast cancer.

In lymph-node negative breast cancer patients, the high score group was associated with a 5.36 fold increased risk compared with low score group. However, because of wide 95% CIs in these categorical analyses, these results should be interpreted with cautions. Yet the 95% CIs became narrower when examining analyses between ordinal value of risk group and DFS (HR, 2.21; 95% CI 1.45-3.35; *P* < 0.001). In our study, neither lipocalin-2 nor MMP-9 level correlated with lymph-node status. Assuming the cooperative role of lipocalin-2 and MMP-9 in tumor progression, poor prognosis associated with high levels of both markers could be attributed to the enhanced activity of MMP-9 by lipocalin-2 and subsequent molecular path related with tumoral invasiveness and diffusion in lymph-node negative status. To date, axillary lymph-node status remains the most powerful predictor for recurrence and survival of primary breast cancer. Nevertheless, approximately one-third of women with node negative breast cancer develop distant metastases 10 years after local therapy [45-47]. Thus, newer prognostic factors are urgently required to identify meaningful high risk subgroups within lymph-node negative breast cancer patients who may benefit from adjuvant therapy regimens. Although the hazard ratios of around 2 obtained from the combined risk score based on the overall patients are not big enough to impact on clinical practice, our result suggests that the combined score of both markers might have potential predictive value for metastasis and recurrence of patients with lymph-node negative breast cancer. However, these results are speculative and need to be confirmed in independent and large number of subjects to evaluate the external validity.

Our study has several limitations. We collected and measured both markers in a single manner, thus could not completely characterize the profiles of both markers accompanied by treatment response and subsequent survival. In addition, due to the relatively short term follow-up time, we could not assess the relationship between both markers and long term survival related to mortality of breast cancer. Thus these results must be interpreted cautiously and need to be confirmed in large prospective trials with serial measurements. In addition, it is worthwhile to investigate if surgical treatment of tumor results in subsequent clearing of both markers in serum assuming that the second primary cancer or recurrent breast cancer is causative for the elevated lipocalin-2 and MMP-9.

## Conclusions

In conclusion, to our knowledge, this is the only study that has evaluated the prognostic role of preoperative serum lipocalin-2 and combined effect of lipocalin-2 and MMP-9 in patients with operable breast cancer suggesting that the elevated levels of lipocalin-2 and MMP-9 are associated with reduced breast cancer survival.

## Abbreviations

aHR, Adjusted hazard ratio; BMI, Body mass index; CI, Confidence intervals; DFS, Disease-free survival; EMT, Epithelial-mesenchymal transition; ER, Estrogen receptor; HR, Hazard ratio; LCN2, Lipocalin-2; MDD, Minimum detectable dose; LN, Lymph-node negative; LN +, Lymph-node positive; MMP-9, Matrix metalloproteinase-9; NGAL, Neutrophil gelatinase-associated lipocalin; PR, Progesterone receptor; SD, Standard deviations.

## Competing interests

The authors have declared no conflicts of interest.

## Author’s contributions

DK, DYN, and SHA were PIs for each of the participating cooperative groups of the Seoul Breast Cancer Study (SeBCS). HS, JYC, SAL, KML, SKP, KYY and DK were involved in conception and design of the study and participated in the discussion and interpretation of the results. HS participated in data analysis and writing of the manuscript. JYC, SH, SJ and MS contributed to statistical analyses and helped to draft the manuscript. YL participated in data collection and sample selection. All authors have read and approved the final manuscript.

## Pre-publication history

The pre-publication history for this paper can be accessed here:

http://www.biomedcentral.com/1471-2407/12/193/prepub

## Supplementary Material

Additional file 1 **Table S1.** Serum levels of lipocalin-2 and MMP-9 were compared between breast cancer cases ( *N* = 303) and age and sex matched healthy controls ( *N* = 74). **Table S2.** The predictive accuracy of model using combined score of Lipocalin-2 and MMP-9 and other prognostic factors. Click here for file
