# Supplementary material for: The association between the preoperative serum levels of lipocalin-2 and matrix metalloproteinase-9 (MMP-9) and prognosis of breast cancer
Source: BMC Cancer. 2012 May 28;12:193. doi: 10.1186/1471-2407-12-193 (PMC3479006; doi:10.1186/1471-2407-12-193)
Supplement: Additional file 1 — Table S1. Serum levels of lipocalin-2 and MMP-9 were compared between breast cancer cases ( N = 303) and age and sex matched healthy controls ( N = 74). Table S2. The predictive accuracy of model using combined score of Lipocalin-2 and MMP-9 and other prognostic factors. [file 1471-2407-12-193-S1.doc]

| **Supplementary Table 1.** Serum levels of lipocalin-2 and MMP-9 were compared between breast cancer cases (*N* = 303) and age and sex matched healthy controls (*N* = 74). | | | | | | | | |
| --- | --- | --- | --- | --- | --- | --- | --- | --- |
|  | Controls (*N* = 74) | |  | Cases (*N* = 303) | |  | *P*^a^ | *P*^b^ |
|  | median (range) | mean (SD) |  | median (range) | mean (SD) |  |
| Age | 44 (40-70) | 48.3 (8.04) |  | 45 (21-77) | 46.6 (10.69) |  | 0.13 | 0.12 |
| BMI (kg/m^2^) | 22.75 (17.9-32) | 23.2 (2.83) |  | 23.2 (16.8-35.0) | 23.3 (3.17) |  | 0.77 | 0.79 |
| Lipocalin-2 (ng/ml) | 86.9 (42.7-187.1) | 93.0 (29.9) |  | 79.3 (2.63-772.3) | 98.3 (79.3) |  | 0.12 | 0.36 |
| MMP-9 (ng/ml) | 44.6 (18.6-293.2) | 62.8 (55.1) |  | 52.4 (0.15-397.1) | 70.5 (64.3) |  | 0.38 | 0.34 |

^a^ *P*-value calculated from Wilcoxon signed rank test.

^b^ *P*-value calculated from Student’s t-test.

**Supplementary Table 2.** The predictive accuracy of model using combined score of Lipocalin-2 and MMP-9 and other prognostic factors.

| Model | *N* | ROC Area | SE | 95% CIs |
| --- | --- | --- | --- | --- |
| combined score^a^ | 303 | 0.568 | 0.036 | 0.50-0.64 |
| combined score^b^ | 303 | 0.570 | 0.036 | 0.50-0.64 |
| combined score + other covariates^c^ | 298 | 0.740 | 0.031 | 0.68-0.80 |
| combined score + other covariates^b,c^ | 298 | 0.711 | 0.032 | 0.65-0.78 |
| ^a^ Fitted with Cox proportional hazard regression model including combined score (continuous) only  ^b^ Fitted with prediction error (PE) adjusted model using 10-fold cross validation.  ^c^ Fitted with Cox proportional hazard regression model including combined score (continuous), TNM stage (I, II, and III), ER status (positive and negative), radiation therapy (yes and no) and BMI (< 25 and ≥25 kg/m^2^). | | | | |
